# A differential DNA methylome signature of pulmonary immune cells from individuals converting to latent tuberculosis infection

**DOI:** 10.1038/s41598-021-98542-3

**Published:** 2021-09-30

**Authors:** Lovisa Karlsson, Jyotirmoy Das, Moa Nilsson, Amanda Tyrén, Isabelle Pehrson, Nina Idh, Shumaila Sayyab, Jakob Paues, Cesar Ugarte-Gil, Melissa Méndez-Aranda, Maria Lerm

**Affiliations:** 1 Division of Inflammation and Infection, Lab 1, Floor 12 Department of Biomedical and Clinical Sciences, Faculty of Medicine and Health Sciences, Linköping University, 58185 Linköping, Sweden; 2 Division of Infectious Diseases, Department of Biomedical and Clinical Sciences, Faculty of Medicine and Health Sciences, Linköping University, 58185 Linköping, Sweden; 3 Facultad de Medicina, Instituto de Medicina Tropical Alexander Von Humboldt, Universidad Peruana Cayetano Heredia, Lima, Peru; 4 Facultad de Ciencias y Filosofía, Laboratorio de Investigación en Enfermedades Infecciosas, Universidad Peruana Cayetano Heredia, Lima, Peru

**Keywords:** Immunology, Microbiology, Diseases, Medical research

## Abstract

Tuberculosis (TB), caused by *Mycobacterium tuberculosis,* spreads via aerosols and the first encounter with the immune system is with the pulmonary-resident immune cells. The role of epigenetic regulations in the immune cells is emerging and we have previously shown that macrophages capacity to kill *M. tuberculosis* is reflected in the DNA methylome. The aim of this study was to investigate epigenetic modifications in alveolar macrophages and T cells in a cohort of medical students with an increased risk of TB exposure, longitudinally. DNA methylome analysis revealed that a unique DNA methylation profile was present in healthy subjects who later developed latent TB during the study. The profile was reflected in a different overall DNA methylation distribution as well as a distinct set of differentially methylated genes (DMGs). The DMGs were over-represented in pathways related to metabolic reprogramming of macrophages and T cell migration and IFN-γ production, pathways previously reported important in TB control. In conclusion, we identified a unique DNA methylation signature in individuals, with no peripheral immune response to *M. tuberculosis* antigen who later developed latent TB. Together the study suggests that the DNA methylation status of pulmonary immune cells can reveal who will develop latent TB infection.

## Introduction

Tuberculosis (TB) is a major global health concern, ranked as one of the top 10 causes of death worldwide and estimated to be responsible for 1.2 million deaths per year^1^. TB is caused by the facultative intracellular bacteria *Mycobacterium tuberculosis* and one-fourth of the world’s population is estimated to be infected making *M. tuberculosis* is one of the most successful pathogens known. *M. tuberculosis* is air-born and enters the lung where the bacteria are internalized by alveolar macrophages (AMs) through phagocytosis. The “checkpoint model” can be used to describe the following immunological events post infection^2,3^. At the first checkpoint, to establish an infection, *M. tuberculosis* needs to evade elimination by AMs. *M. tuberculosis* has developed several strategies to manipulate the host immune response to extend its survival in the phagocytes. The pathogen can arrest maturation of the phagolysosome and direct phagocytes to necrosis, which is prerequisite for the bacterium to spread. In some individuals, referred to as *early clearers*, the pathogen is successfully cleared by innate immune mechanisms at this initial stage of infection^4–6^. If the pathogen is replicating and cannot be cleared by the innate immune system, the second checkpoint is reached; activation of the adaptive immune system. When the infection is controlled by the adaptive immune system, asymptomatic latent TB infection has developed. From this point, there is a 5–10% lifetime risk of progression to active TB, as a result of inadequate immune control, which is the third checkpoint for *M. tuberculosis*^7–10^. The Interferon-Gamma Release Assay (IGRA) is an immunological test used to confirm whether a subject has been exposed to *M. tuberculosis* based on peripheral T cell release of interferon-γ (IFN-γ) in response to *M. tuberculosis* antigens^11^. The role of epigenetics in TB immune responses is emerging. Several studies have described the concept of developing trained immunity through epigenetic reprogramming, leading to proper orchestration of gene expression upon re-exposure to a pathogen or pathogen-derived products^3,12,13^*.* DNA methylation, histone modifications, and regulation of small RNAs are considered as the important players in the regulation of epigenetic modifications^14^. We and others have described the reprogramming of DNA methylation patterns in peripheral blood mononuclear cells (PBMCs) after exposure to live attenuated *Mycobacterium bovis* through the *Bacillus Calmette Guérin* (BCG) vaccination^15,16^. Further, we have demonstrated that differences in these DNA methylation patterns affect the efficacy of the macrophages to kill *M. tuberculosis *in vitro^17^*.* A wealth of literature focuses on adaptive immune responses in peripheral blood, but since *M. tuberculosis* primarily infects the lung, it is relevant to address pulmonary immunity, including alveolar T cells and AMs^18,19^. In this study, we have increased our focus on the pulmonary-resident immune cells and further investigated DNA methylomes of AMs and alveolar T cells in a cohort of medical students with a previously reported increased risk of *M. tuberculosis* exposure^20^. The aim of the study was to investigate epigenetic modifications in the pulmonary immune cells pre- and post-TB exposure in a natural setting. We hypothesized that recent exposure to *M. tuberculosis* in the lung compartment would induce epigenetic alterations in AMs and alveolar T cells. The AMs and alveolar T cells were isolated by sputum induction^21^ and using reduced representation of bisulfite sequencing (RRBS) the DNA methylome was investigated. We identified an altered DNA methylation profile in the pulmonary immune cells of the subjects that later developed latent TB infection as compared to those who tested negatively for latent TB throughout the study. Notably, this DNA methylation profile was identified in the immune cells before a latent TB infection could be detected with the IGRA test. The differentially methylated genes (DMGs) identified in the subjects developing latent TB infection were over-represented in the pentose phosphate pathway in AMs and in IFN-γ signaling and migration in the alveolar T cells.

## Results

### Study design and cohort

**Figure 1:**
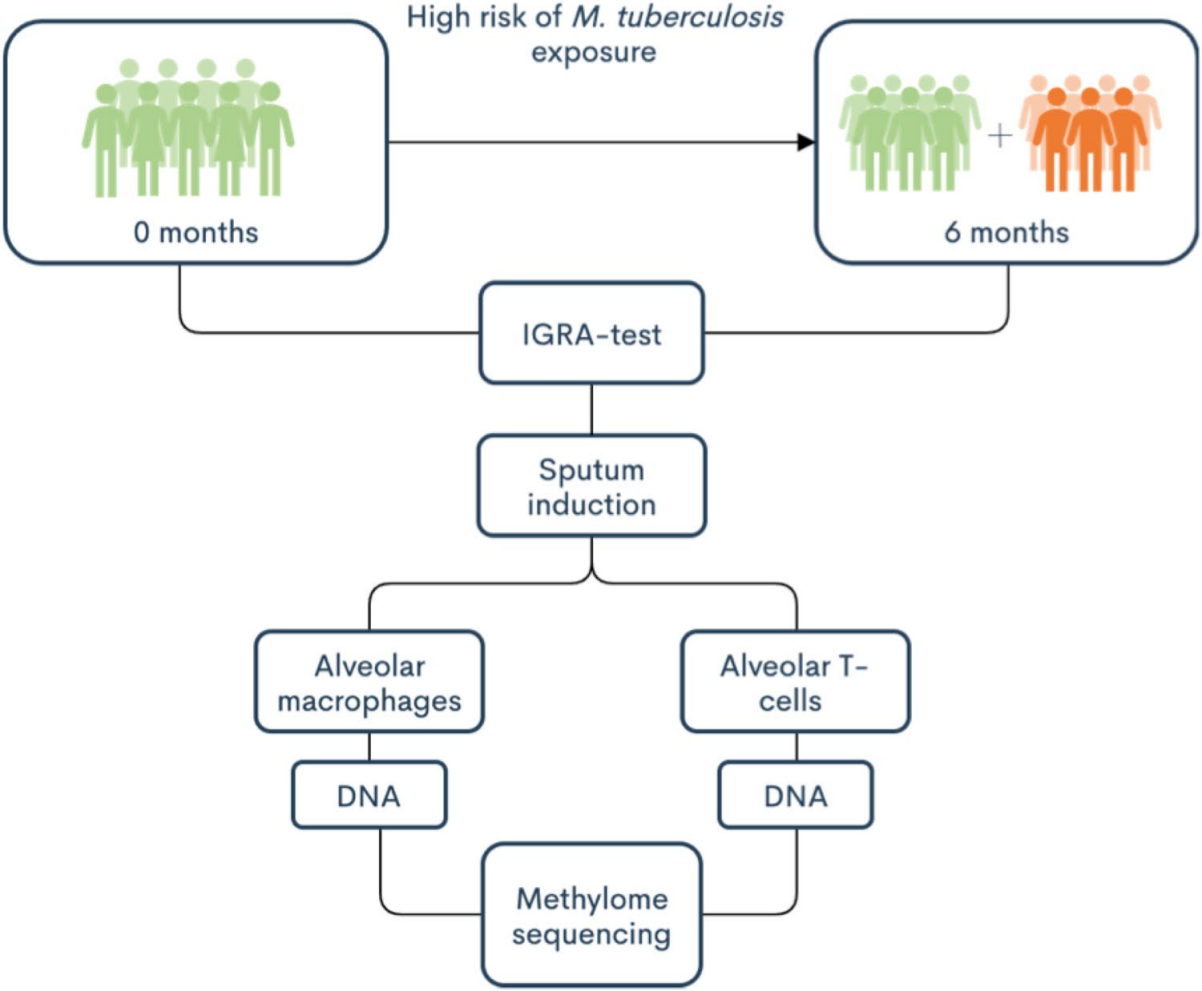
Flow chart of the study design. Study subjects donated sputum and blood samples at 0 and 6 months, which corresponded to before and after clinical rotations at departments with high-risk of *M. tuberculosis* exposure at hospitals in Lima, Peru. Interferon-Gamma Release Assay (IGRA) tests were taken to confirm TB-infection. Alveolar macrophages (AMs) and alveolar T cells were isolated from sputum samples. DNA was extracted and the samples were sequenced with reduced representation bisulfite sequencing (RRBS) for methylation analysis.

Medical students were invited to participate in the study and donated sputum samples before (referred here as 0 months) and after (referred here as 6 months) clinical rotations in departments with a high-risk of *M. tuberculosis* exposure. A schematic overview of the study design is represented in Fig. 1. Demographic information of the study subjects and IGRA results are shown in Table 1. In elaborate questionaries the health status of the study participants was evaluated, and no study participant had any conditions of immunosuppression or severe illnesses during the study. One study subject was borderline IGRA-positive^22^ (IGRA_pos_) already at inclusion (0 months), IGRA results presented in supplementary Table S1. Two study subjects developed latent TB infection during the study as demonstrated with a positive IGRA test at follow-up (6 months, referred to as IGRA converters in the following text) (Table S1).

**Table 1:** Demographics of study subjects. A total of 15 participants were included in the study. Interferon-Gamma Release Assay (IGRA) test confirmed IGRA conversion in two study subjects. ^✢^ shows the range of the data.

Characteristics	Participants (*n* = *15*)
Sex (male and female)	8/7
Age (years)	22.7 (21–29)^✢^
Weight (kg)	70.5 (48.5–101)^✢^
Height (cm)	168.8 (159–182)^✢^
BMI (kg/m^2^)	24.6 (17.2–34.5)^✢^
IGRA result 0 months (negative and positive)	14/1
IGRA result 6 months (negative and positive)	12/3

### DNA methylation patterns in alveolar macrophages and alveolar T cells before and after TB exposure distinguish IGRA converters

**Figure 2:**
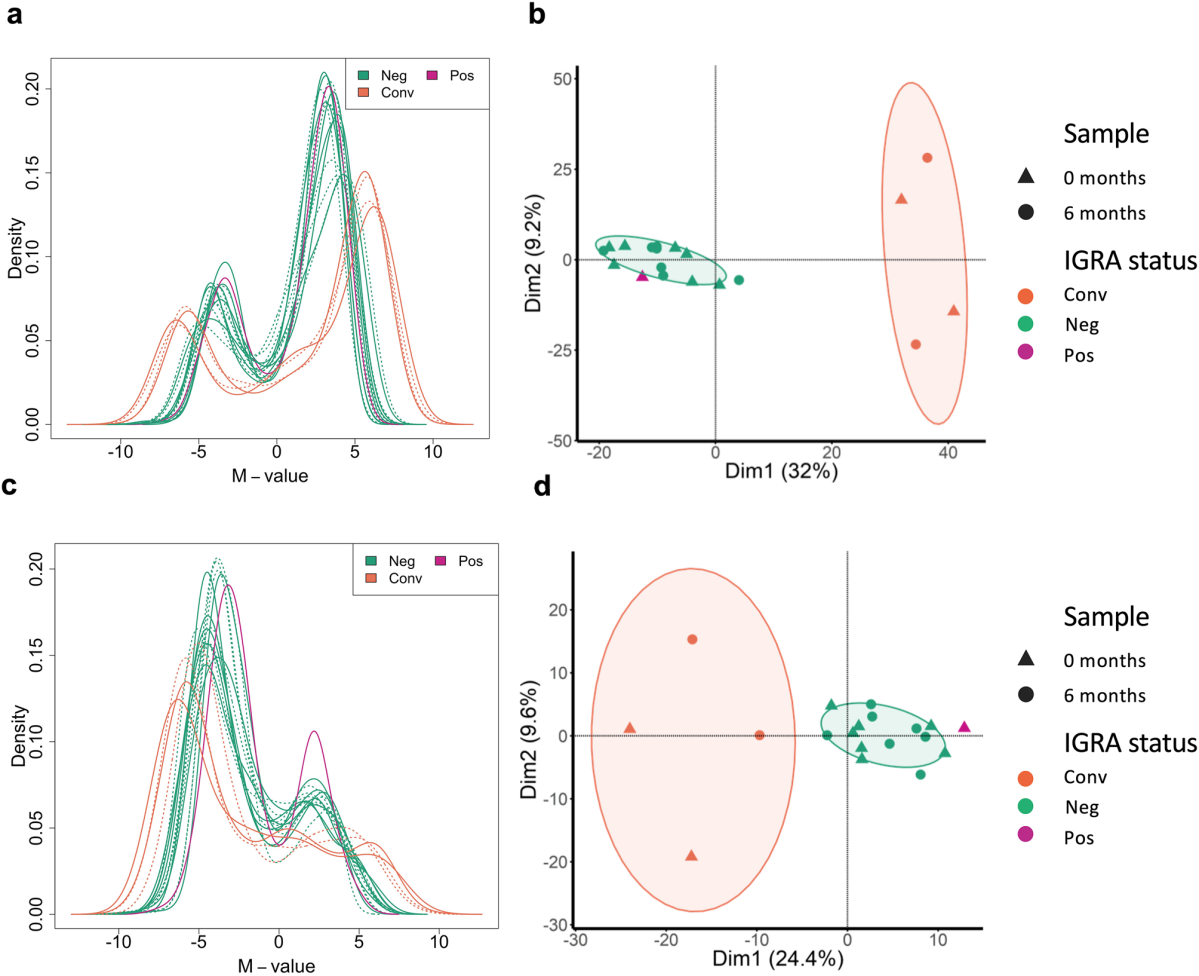
Unique distribution of genomic DNA methylation discriminates IGRA-converting individuals. Density plots of the distribution of M-values from CpG-sites identified in (**a**) alveolar macrophages (AMs) and (**c**) alveolar T cells. Full line represents samples collected at 0 months and dotted lines samples collected at 6 months. The IGRA status is explained by the color of the line, negative (green), converter (orange) and positive (pink). The Principal Component Analysis (PCA) of the methylation data from (**b**) AMs and (**d**) alveolar T cells. The timepoint of sample collection is explained by shape, triangle for 0 months and dot for 6 months, the IGRA-status is explained by color. The ellipses represent the 70% confidence interval (CI) in the dataset. AMs (*n* = 19), alveolar T cells (*n* = 19).

**Figure 3:**
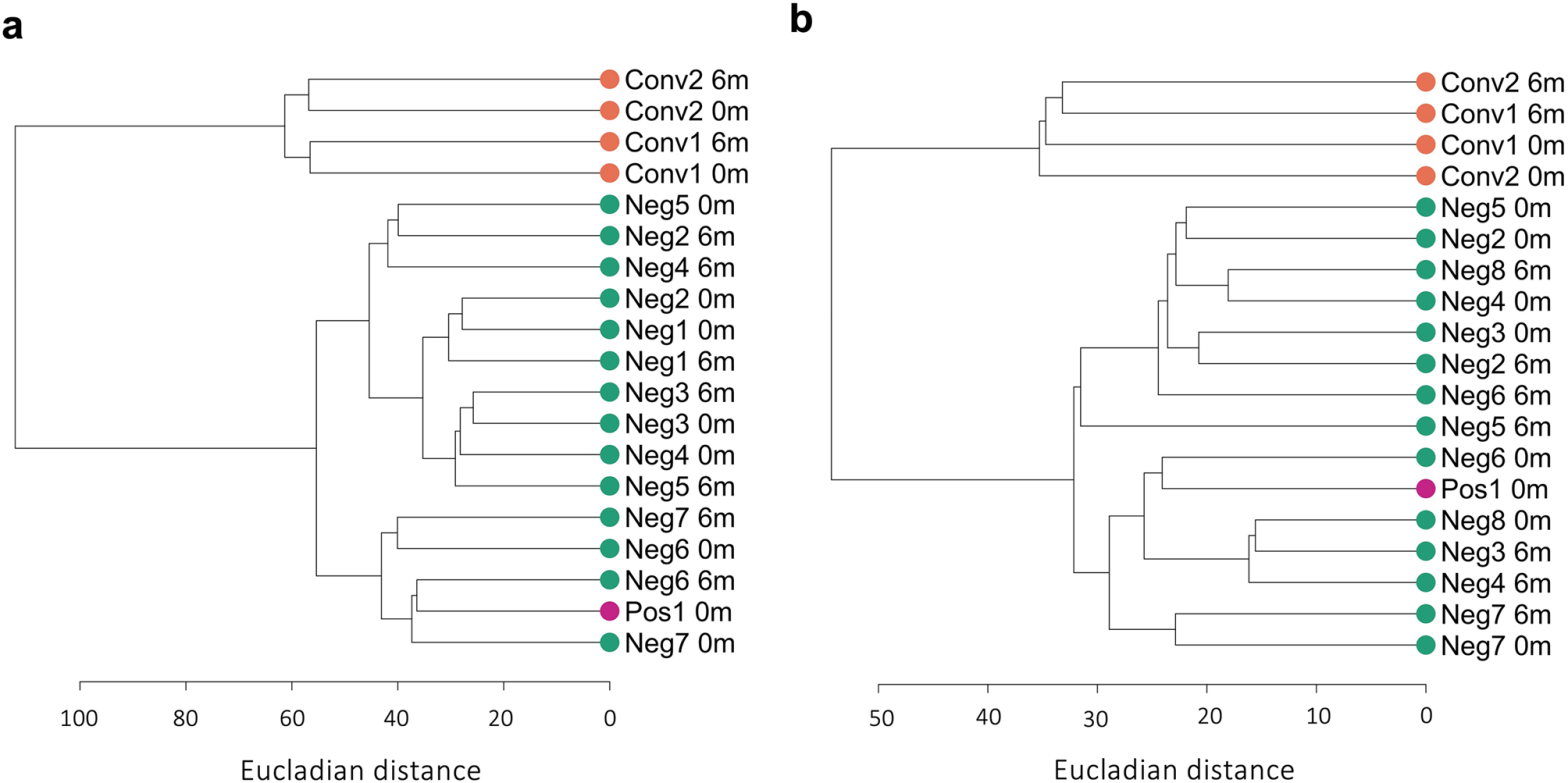
Hierarchical clustering analysis separating IGRA-converting individuals. Unsupervised hierarchical clustering dendrogram applying the Euclidian distance matrix calculation and Ward D2 method. The dendrograms show the clustering of methylome data from (**a**) alveolar macrophages (AMs). and (**b**) alveolar T cells. The scale represents the Euclidean distance. AMs (*n* = 19), alveolar T cells (*n* = 19).

**Figure 4:**
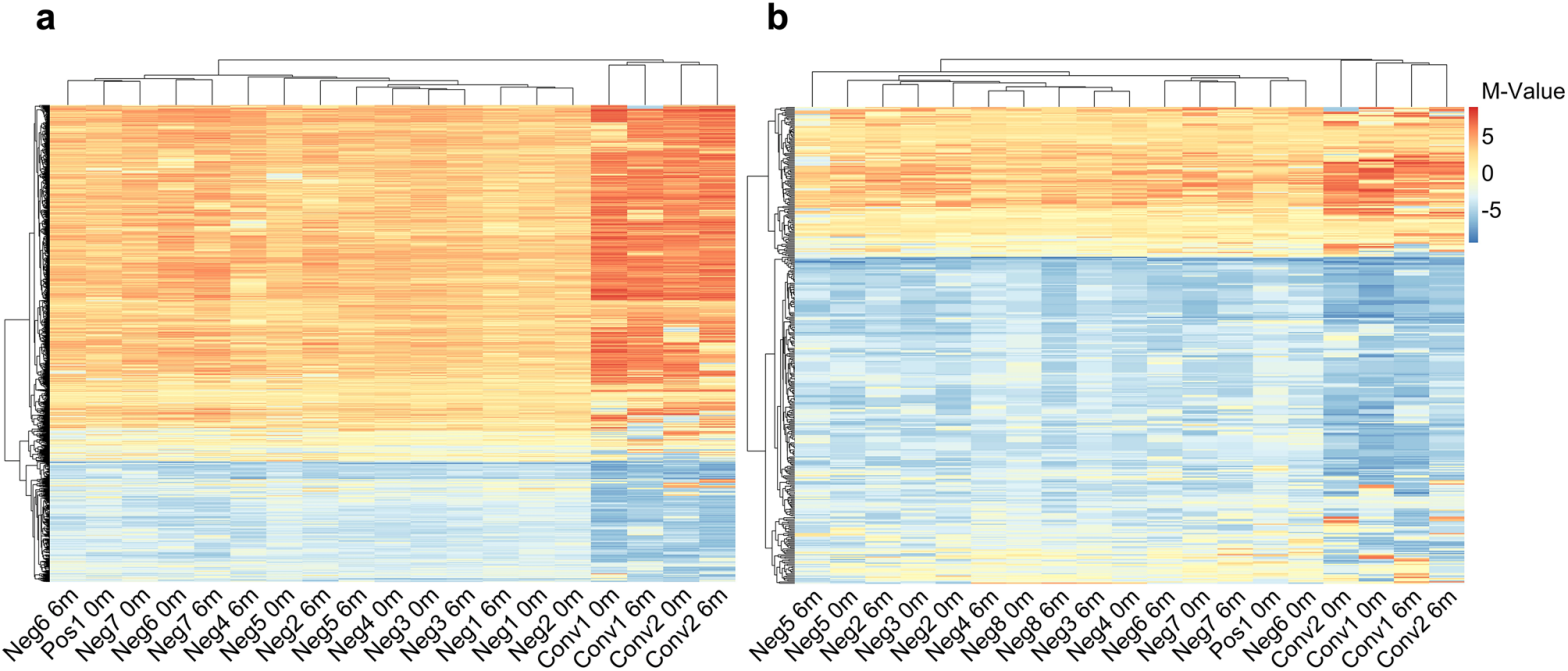
Heatmaps reveal a different overall DNA methylation pattern in IGRA-converting study subjects. (**a**) Heatmap of all CpG-sites (1186) identified in the alveolar macrophages (AMs) of each study subject. (**b**) Heatmap of all CpG-sites (404) identified from the alveolar T cells. The color bar represents the M-value scale ranging from − 10 (blue) to 10 (red). AMs (*n* = 19), alveolar T cells (*n* = 19).

To investigate the epigenetic changes in the pulmonary immune cells over time, we isolated DNA from the AMs and alveolar T cells^21^ collected at 0 and 6 months and performed Genome-wide Reduced Representation Bisulfite sequencing (RRBS). After filtering the data as described in the method section, we identified a total of 1,186 CpG-sites with ≥ 5 reads in the 19 samples from the AMs and 404 CpG-sites in the 19 samples from the alveolar T cells. To get an overview of the global DNA methylation distribution in the pulmonary immune cells, we made density plots of the M-values (the log_2_ ratio of the intensities of methylated versus unmethylated CpG-sites) obtained from CpG-sites in the AMs (1186 CpG-sites) and the alveolar T cells (404 CpG-sites), shown in Fig. 2a,c. We identified a clear difference in the methylation distribution between the cell types, AMs and alveolar T cells. The density plot showed a homogenous distribution of DNA methylation in the samples from the IGRA_neg_ study subjects collected at both at 0 and 6 months. The sample from one study subject, that was borderline IGRA_pos_ at inclusion, followed the same global DNA methylation distribution. Whereas in the IGRA-converting study subjects, we identified a different global DNA methylation profile with more hyper- and hypomethylated CpG-sites as well as different peak densities. Notably, the sample collected at 6 months (after IGRA conversion) displayed a similar DNA methylation profile as the sample collected at inclusion. The results coincided in both the AMs and the alveolar T cells. In a subset of three donors transcriptome data was obtained and we extracted the individual expression counts from the transcriptome data using the 1,186 identified CpG-sites. The spearman’s rank analysis revealed negative correlations between the methylation and transcriptome data for each individual implying the hypermethylated genes are down-regulated and vice versa (Figure S1 a,b). To further explore the data, we performed a principal component analysis (PCA) which revealed a distinct group formation from the IGRA converters (based on the PC1 (Dim1) with 70% C.I.), including both the samples collected before and after the IGRA conversion (Fig. 2b,d). The borderline IGRA_pos_ individual on the other hand clustered with the data obtained from IGRA_neg_ study subjects. A variance decomposition analysis between the principal components and known possible confounding variables including age, BMI, sample collection timepoint, gender, batch and IGRA status showed that in the first PCs IGRA status and BMI explained a substantial portion of the variance in both cell types (Figure S2a,b), no significant difference in the BMI of the groups was measured with a Mann–Whitney U test (Figure S2c). We proceeded with a hierarchical clustering analysis by applying the Euclidean similarity/dissimilarity matrix calculation using the M-value (Fig. 3a,b). In line with the PCA results, the data from the IGRA converters formed a separate cluster, including both the sample collected before and after IGRA conversion. The same cluster separation was found in both the samples from the AMs and from the alveolar T cells. To visualize the methylation status in the 1186 and 404 CpG-sites from the AMs and alveolar T cells respectively, we created heatmaps (Fig. 4a,b), demonstrating a clear difference in the overall methylation between IGRA converters and IGRA_neg_ study subjects.

### High number of strong DMCs identified in IGRA-converting individuals

**Figure 5:**
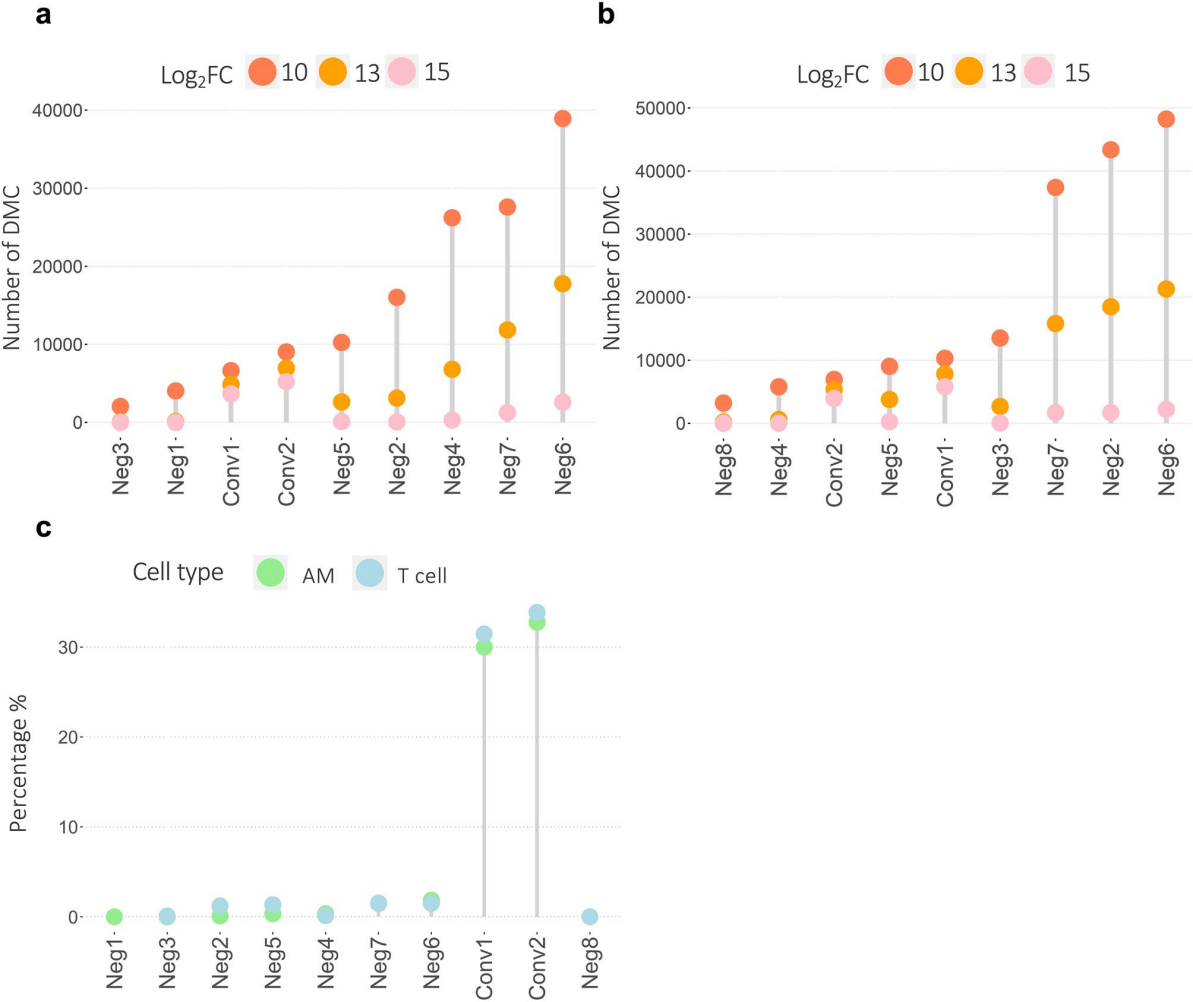
Profound DNA methylome alterations identified in IGRA-converting study subjects. Lollipop plots showing the number of differentially methylated CpG-sites (DMCs) identified in the (**a**) alveolar macrophages (AMs) and (**b**) alveolar T cells of each study subject at (|log_2_FC| cutoff at > 10, 13 and 15. (**c**) The percentage of DMCs with a |log_2_FC|> 15 compared to the total number of DMCs (|log_2_FC|> 5, BH adj *p *value > 0.01) identified in each study subjects’ AMs and alveolar T cells.

To further explore the epigenetic reprogramming that occurred between the two time points, 0 and 6 months, we identified the differentially methylated CpG-sites (DMCs) with the strict filtering criteria (|log_2_ Fold Change (log_2_FC)|> 5 and Benjamini-Hochberg (BH) adjusted *p *value < 0.01) in each study subject. The DMCs were divided into three different cutoff levels: |log_2_FC| 10, 13 and 15 hyper- or hypomethylation in order to understand the distribution of DMCs based on the level of change. The number of DMCs in the different cutoff levels for each study subject is presented in Fig. 5a (AMs) and 5b (T cells). A high number of DMCs with a |log_2_FC|> 15 was identified in the IGRA converters in both cell types. To normalize the results to account for the different number of total DMCs identified (|log_2_FC|> 5, BH adj. *p *value < 0.01) we looked at the percentage of DMCs with a |log_2_FC|> 15. The percentage of DMCs with a |log_2_FC|> 15 for each cell type and study participant is shown in Fig. 5c.

### IGRA-converting individuals undergo similar epigenetic changes in DNA methylation

**Figure 6:**
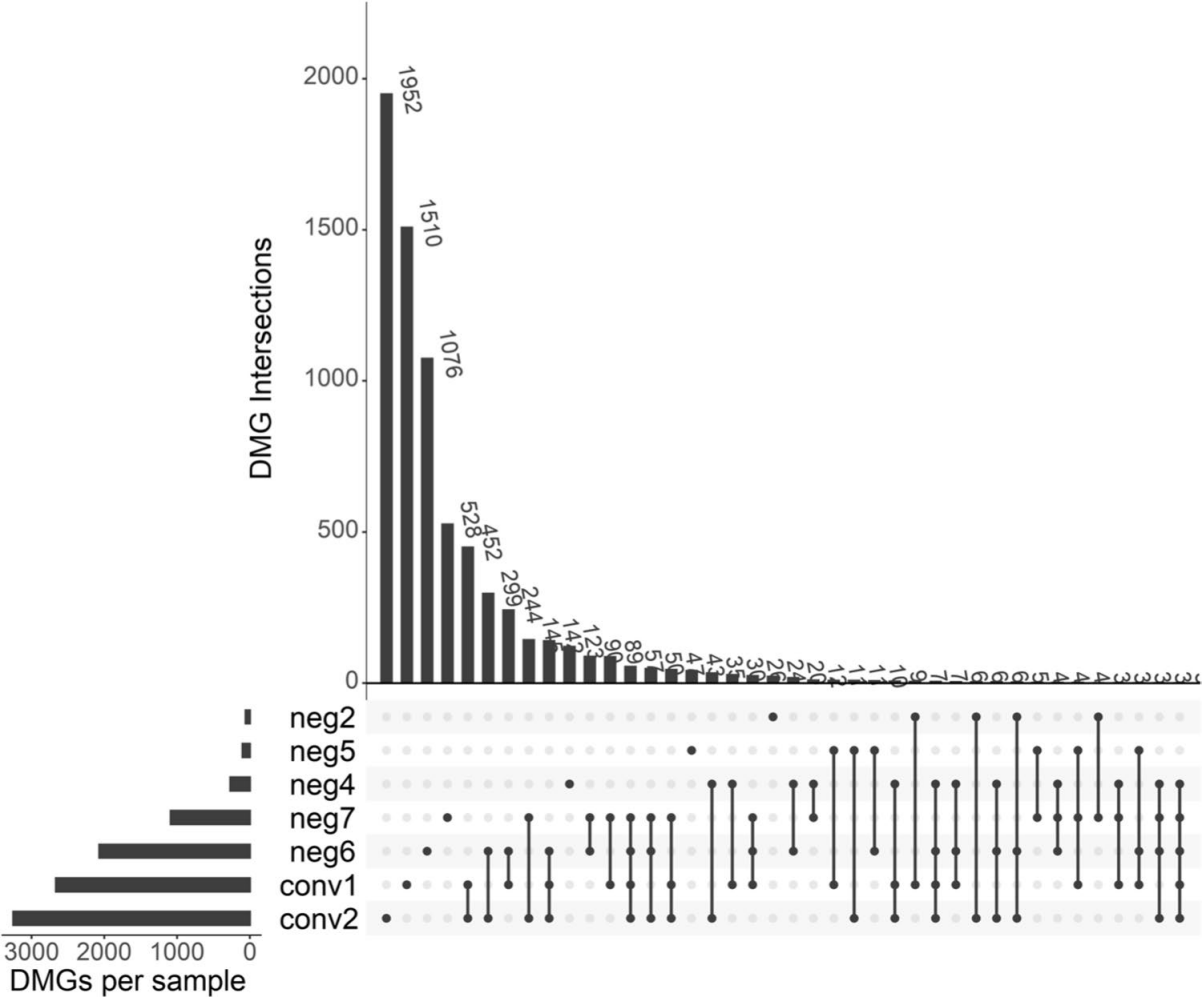
IGRA converters undergo unique DNA methylation changes during IGRA conversion. UpSet plot showing the intersects of the differentially methylated genes (DMGs) identified in each study subjects’ alveolar macrophages.

The DMCs were annotated to the official (HUGO Gene Nomenclature Committee approved) Gene Symbols for further analysis and is referred to as DMGs in the following text. To identify the overlap in the DMGs with a |log_2_FC|> 15 between the study subjects, a Venn analysis was performed and presented in UpSet plots. The IGRA converters shared the largest intersection in both cell types, 452 DMGs in the AMs (Fig. 6) and 471 DMGs in the alveolar T cells (Supplementary Figure S3). 32 of these DMGs were overlapping between the cell types. To filter out unspecific changes we selected the DMGs that changed in the same direction, hyper- or hypomethylated, in both IGRA converters. We identified 280 (128 DMGs were hypermethylated and 152 hypomethylated) and 281 (159 DMGs were hypermethylated and 122 hypomethylated) DMGs that became hypo- or hypermethylated, in both IGRA converters in the AMs and in the alveolar T cells, respectively.

### IGRA converters’ DMGs are over-represented in pathways related to metabolic reprogramming, T cell migration and IFN-γ production

In an over-representation analysis (ORA)^23^ using the PANTHER database^24^, the identified DMGs from the AMs unique to the IGRA converters were shown to be over-represented in the pentose phosphate pathway and the Ras pathway (Table 2). In an ORA of the DMGs from the alveolar T cells unique to the IGRA converters we identified enrichment in the muscarinic acetylcholine receptors 1–4 pathway and β1- and β2 adrenergic receptor signaling pathway (Table 3).

**Table 2:** Over-representation analysis of DMG intersect from IGRA converter’s alveolar macrophages. Over-representation analysis using PANTHER pathways of the differentially methylated genes (DMGs) intersect identified in the alveolar macrophages of the IGRA converters. ‘Gene Set’ is PANTHER pathway accession. ‘FDR’ is BH adjusted *p *values. ‘Ratio’ is the enrichment ratio.

Gene Set	Description	Ratio	*p *value	FDR
P02762	Pentose phosphate pathway	20.981	0.004	0.41
P04393	Ras pathway	3.596	0.049	1

**Table 3:** Over-representation analysis of DMG intersect from IGRA converter’s alveolar T cells. Over-representation using PANTHER pathways of the differentially methylated genes (DMGs) intersect identified in the T cells of the IGRA converters. ‘Gene Set’ is PANTHER pathway accession. ‘Ratio’ is the enrichment ratio. ‘FDR’ is BH adjusted *p* values.

Gene set	Description	Ratio	*p *value	FDR
P00042	Muscarinic acetylcholine receptors 1 and 3 signaling pathway	6.34	0.003	0.35
P04377	Beta 1 adrenergic receptor signaling pathway	6.08	0.012	0.44
P04378	Beta 2 adrenergic receptor signaling pathway	6.08	0.012	0.44
P00043	Muscarinic acetylcholine receptors 2 and 4 signaling pathway	4.8	0.022	0.44

## Discussion

In this study we investigated the genome-wide DNA methylation in the pulmonary immune cells in individuals at high risk of exposure to *M. tuberculosis*. DNA methylation has been shown to play an important role in the innate immune systems response to mycobacteria by us and others^15,25–28^, however, it has not yet been longitudinally studied in healthy individuals at risk of TB exposure. Two of the study subjects developed latent TB infection during the course of sample collection as demonstrated by IGRA conversion and our analyses identified a distinct genome-wide DNA methylation profile separating these two individuals from those who remained IGRA_neg_ throughout the study. This DNA methylation profile, which, in contrary to our expectation, was present already at inclusion, was characterized by more hypo- and hypermethylated CpG-sites. From these observations, we propose the following hypotheses: (i) the IGRA converters were exposed to TB via the airways already before inclusion and the exposure had not yet developed into circulating T cell memory (resulting in a negative IGRA test) but had caused reprogramming of the DNA methylome of AMs; (ii) a different, *inherent* genomic DNA methylation profile as observed in the IGRA converters predispose for IGRA conversion in a high-endemic setting.

According the first hypothesis, the observed DNA methylation pattern represents a normal, protective response to TB exposure (as part of the first ‘checkpoint’), which however was breached by the infection and therefore urged the host to induce an adaptive immune response (the second ‘checkpoint’)^2,3^. If the second hypothesis applies, a limited capacity for inducing a host-protective epigenetic reprogramming could cause susceptibility and predisposition for developing latent TB infection. There is a large heterogeneity in the human susceptibility to develop latent or active TB upon exposure and there is limited knowledge in the determining factors^29^, but larger studies could investigate the correlation between DNA methylation patterns and TB susceptibility. It is also possible that the distinct DNA methylation pattern observed could be affected by *M. tuberculosis* manipulating the host cells epigenome^26,30,31^.

The two IGRA converters underwent similar epigenetic changes between the timepoints during conversion to IGRA positivity. There was a large overlap in DMGs in the IGRA converters in both cell types. The DMG intersect unique to the IGRA converters AMs showed over-representation in the pentose phosphate pathway which has previously been described to be critical in *M. tuberculosis* infected macrophages^32,33^. Infected macrophages become dependent on both glycolysis and the pentose phosphate pathway to fulfill the metabolic and bioenergetic requirements for production of cytokines, chemokines and reactive oxygen species (ROS)^32–34^. The DMGs unique to the IGRA converters’ alveolar T cells showed over-representation in the pathway for β-adrenergic and muscarinic acetylcholine receptor signaling. A functional role between β2 adrenergic receptor (β2AR) activation and IFN-γ and Tumor necrosis factor (TNF) production in T helper 1 (Th1) cells has been established in several studies, activation of β2AR by the neurotransmitter norepinephrine (NE) decreases the IFN-γ expression in Th1 cells^35–37^. However, in murine models, NE has been shown to induce rapid differentiation of naïve T cells into Th1 cells producing high levels of IFN-γ which have been correlated to early TB infection^38,39^. Polymorphisms in the β2AR have also been reported to be associated with TB^40^. The cholinergic system also has a functional role in the T cells. T cells express both muscarinic and nicotinic acetylcholine (ACh) receptors and expression of the enzyme choline acetyltransferase (ChAT) is induced in T cells during infection, T cell derived ACh is involved in T cells migration to tissues^41–43^.

One study subject was borderline IGRA positive at inclusion and this subject followed the same DNA methylation distribution and was clustering with the IGRA_neg_ study subjects. The kinetics of the IFN-γ-reaction in relation to point of infection is not elucidated and after TB treatment the peripheral T cell memory will persist for up to 15 months^44,45^. According to our analysis, the epigenome of the pulmonary immune cells in this individual was more similar to that of healthy individuals, possibly indicating a historical exposure to TB and that the epigenome profile has returned to baseline.

We identify different clinical implications of the findings presented here. If the DNA methylation profile observed in the IGRA converters is an early result of *M. tuberculosis* infection, this could have implications as a diagnostic tool for identification of individuals that are developing latent TB infection before any of the currently available diagnostic methods. However, if the pattern we identified is not a result of bacterial infection but rather an epigenomic dysregulation predisposing individuals to convert upon exposure, this global DNA methylation pattern could have implications in identifying risk-groups who are more prone to convert upon exposure.

We recognize that this study was performed on a small number of study subjects and further investigation in larger cohorts is needed to elucidate the results presented here. We also acknowledge the limitations of the study design, we included a cohort of study subjects with risk of *M. tuberculosis* exposure, but no definite measurement of exposure. The observations made with IGRA converters were limited to two study subjects. However, we recently published a study that supports the finding of a differential DNA methylation pattern that can distinguish individuals with latent TB from IGRA-negative individuals in a similar high endemic setting in Lima, Peru^46^ (MedRxiv pre-print, 2021). We also recently showed that *M. tuberculosis* exposure-induced epigenetic reprogrammings are more profound in pulmonary immune cells as compared to PBMCs^47^ (MedRxive pre-print 2021). Here, we highlight the significance of investigating pulmonary immune cells, and the value of investigating DNA methylomes in the same study subjects, longitudinally, before and after IGRA conversion.

## Methods

### Ethical statement

Ethical approval was obtained from Universidad Peruana Cayetano Heredia (UPCH) Institutional Review Board (IRB), No. 103793. The sputum sample collections were performed in accordance with guidelines from the Department of Respiratory Medicine at Linköping University Hospital. The IGRA samples (QuantiFERON® TB-Gold Plus test) were collected and analyzed by medical personnel according to the guidelines at the Instituto de Medicina Tropical Alexander von Humboldt, Universidad Peruana Cayetano Heredia. All participants signed an informed consent.

### Study cohort and design

This was a prospective study aimed to investigate epigenetic DNA methylome alterations in pulmonary immune cells pre- and post-*M. tuberculosis* exposure in a natural setting. We enrolled medical students (*n* = 15) in the fifth or sixth year of medical school at UPCH. The students donated sputum and blood samples before (0 months) and after (6 months) they had clinical rotations in high-risk departments of *M. tuberculosis* exposure. The department of infectious disease and internal medicine including the emergency department were defined as departments with high-risk of *M. tuberculosis* exposure. Sputum was used to isolate immune cells from the lung and the blood was used for IGRA. At inclusion, the participants filled in an individual case report form with demographic information. Participants also filled in an online questionnaire to collect background information before each session at 0 and 6 months. The questionnaire was created in the tool Survey & Report, provided by Linköping University. Samples from 10 individuals (collected at 0 and 6 months for IGRA_neg_ (*n* = *7*) and IGRA converters (*n* = *2*) and at 0 months for IGRA_pos_ (*n* = *1*)) resulted in 19 samples that were selected for each cell type (AMs and alveolar T cells), for the sequencing analysis. The sample selection was based on results in the online questionnaire and on the sample quality with regards to the DNA concentration obtained in the AMs and alveolar T cells at the two sample collections at 0 and 6 months.

### Interferon gamma releasing assay with QuantiFERON® TB-Gold Plus

At inclusion and follow-up, the participants donated blood samples that were used for IGRA with the QuantiFERON® TB-Gold Plus test (SSI Diagnostica, Hillerød, Denmark) according to the manufacturer’s instructions. Tubes were filled and incubated at 37 °C for maximum 24 h and then analyzed with ELISA. The test results are presented in Supplementary Table S1 as the quantification of interferon-γ in international units per ml (IU/ml). There is a dichotomous cut-off (0.35 IU/ml) that defines a positive result, but a borderline range (0.20–0.99 IU/ml) exists^22^.

### Sputum induction

The sputum induction^21^ was performed with an eFlow rapid nebulizer (PARI, Hamburg, Germany) filled with a hypertonic saline solution. The solution was prepared by mixing sterile water (Fresenius Kabi, Stockholm, Sweden) with 4% sodium chloride (B. Braun Medical AB, Stockholm, Sweden). The participants inhaled the solution for 9 min while simultaneously preforming breathing exercises in accordance with instructions from the lung clinic at Linköping University Hospital. The participants were asked to cough deeply to expectorate sputum from the lungs. The expectorates were collected into sterile 50 ml Falcon tubes (Thermo Fisher Scientific, Waltham, US) after each session. The Falcon tubes were kept on ice. The sputum inductions were performed in three replicates.

### Sputum processing and CD3 and HLA-DR positive cell isolation

From the sputum samples, within 2 h, plugs containing pulmonary immune cells^21^ were picked and pooled. The plugs were dissolved by adding (0.1%) dithiothreitol (DTT) (Thermo Fisher Scientific) mixed in phosphate-buffered saline (PBS) (Gibco, Cambridge, UK). This was added in a volume approximately 4 times the collected sputum volume and then vortexed and placed on a tilter with ice for 20 min. The dissolved sputum sample was then filtered through 50 μm cell strainers (Sigma-Aldrich, Saint Louise, US) into a new 50 ml Falcon tube and centrifuged for 5 min at 380 g in 4 °C. The supernatant was discarded, and the pellet was resuspended in (500 µl) of an isolation buffer containing (500 mM) ethylenediaminetetraacetic acid (EDTA), (0.1%) fetal bovine serum (FBS), Ca^2^^+^ and Mg^2^^+^ free PBS (pH 7.4). From the cell pellet we did sequential positive isolation using antibody conjugated magnetic beads. First, we isolated CD3 positive cells to ensure that all T cells were isolated from the cell suspension. Secondly, from the residual cell pellet we isolated HLA-DR positive cells. We first isolate CD3 positive cells to avoid isolating HLA-DR expressing T cells during the second positive isolation. CD3 Dynabeads (25 µl) (Invitrogen Dynabeads®, Life Technologies AS, Oslo, Norway) were washed with (800 µl) PBS and placed in a DynaMag-2 (Thermo Fisher Scientific) for 1 min. The supernatant was discarded, and isolation buffer (800 µl) was added two times for additional washing. The beads were then mixed with the cell sample (500 µl) and incubated for 30 min at 4 °C while tilting. After incubation, the tube was placed in the DynaMag-2, supernatant was removed to be used to isolate HLA-DR positive cells next. The CD3 positive cells were resuspended in (200 µl) PBS.

Secondly, HLA-DR positive cells were isolated. Magnetic Pan Mouse IgG Dynabeads™ (25 µl) (cat no: 11041, Thermo Fisher) were washed with (800 µl) PBS and placed in a DynaMag-2 (Thermo Fisher Scientific) for 1 min. The supernatant was discarded and (800 µl) isolation buffer was added two times for additional washing. Monoclonal HLA-DR antibodies (5 µl) (cat no: 14–9956-82, Thermo Fisher) were added to conjugate the Dynabeads. Tubes were incubated for 40 min, then placed in the DynaMag-2 for 30 s and supernatant removed. (800 µl) isolation buffer was added two times for washing. The tube was placed in DynaMag-2 for 30 s and supernatant removed. The beads were then mixed with the supernatant from the CD3 isolation (500 µl) and incubated for 30 min at 4 °C while tilting. After incubation, the tube was placed in the DynaMag-2, supernatant was discarded, and HLA-DR positive cells were resuspended in PBS (200 µl). CD3 positive cells are referred to as alveolar T cells and HLA-DR positive cells are referred to as alveolar macrophages (AMs).

### DNA and RNA extraction and quantification

DNA and RNA was extracted from the AMs and alveolar T cells with the AllPrep® DNA/RNA Mini Kit (Qiagen, Hilden, Germany) within 4 h from cell isolation. Concentration of DNA and RNA was quantified with a Qubit® 4.0 Fluorometer (Thermo Fisher Scientific), using dsDNA High Sensitivity (HS) Assay Kit or RNA HS Assay kit (Thermo Fisher Scientific). The measurement was performed according to the manufacturer's instructions.

### Reduced representation bisulfite sequencing of DNA from AMs and alveolar T cells

DNA samples were sequenced with Reduced Representation Bisulfide Sequencing (RRBS) at the Bioinformatics and Expression Analysis (BEA) core facility at Karolinska Institute (KI) with Diagenode’s RRBS. The DNA was enzymatically digested, bisulfite-converted, and PCR amplified before ready for Illumina’s HiSeq 2000. The use of the restriction enzyme MspI, which cleaves CCGG from the 5′end, results in shorter sequences to analyze and is therefore cost-effective.

### Transcriptome sequencing of total RNA from AMs

The library was prepared using the SMARTer® Stranded Total RNA Sample Prep Kit—HI Mammalian (Takara Bio Inc, Japan) as per manufacturer’s instructions. The ribosomal RNA was removed, and the remaining RNA was reversely transcribed to cDNA. The cDNA was amplified with specific reverse index primers (Corresponding to TruSeq HT i7 index D701-D712) and forward the index primer (Corresponding to TruSeq HT i5 index primer D502). The concentration of each library was validated with Qubit and RNA HS Assay kit (Thermo Fisher Scientific). One µl of each library were used to analyze average library length with the Agilent 2100 Bioanalyzer and the High sensitivity DNA Chip (Agilent Technologies, USA). 0.5 nM of each library were pooled. The RNA concentrations in nanomolar were calculated using the following equation:

1 $$\frac{{cDNA \left( {\frac{ng}{{\mu l}}} \right) \times 10^{6} }}{{average\, length \left( {bp} \right) \times 660}} = nM$$

A total of 6 samples were sequenced (three samples from the first inclusion and three samples from the second inclusion) and we added a 10% PhiX control in the Illumina NextSeq 550 sequencer (Illumina, US) using the mid output kit (v2.5, 150 cycles) (Illumina). The PhiX control library consists of a characterized bacteriophage genome with a balanced GC (45%) and AT (55%) content. PhiX is used to examine the overall performance of the sequencing. The sequencing was run with a single index, paired-end protocol, both read lengths set to 76 bp according to the manufacturer’s protocol. The sequence result was saved in FASTQ file format.

### Transcriptome data processing and computational analysis

The data quality was checked with FastQC algorithm and showed a phred quality score ≥ 33 in all samples. The raw reads from FASTQ format files were aligned to the Genome Reference Consortium Human Build 38 patch release 13 (GRCh38.p13) using Rsubread (version 3.10) we used the first filtration criteria to set the read length of fragments from 50 to 700 bp to remove unwanted binding sequences. Second filtration was applied to remove the X and Y chromosomes to reduce gender biasness.

### DNA methylation data processing and computational analysis

The RAW files (in fastq format) generated from the RRBS analysis, were quality checked using the fastQC^48^ (v0.11.9). The sequences were trimmed to remove artificially filled-in cytosines at the 3′ end using the TrimGalore (v.0.6.5)^49^ with a phred score cutoff of 20 and quality checked again after trimming. The trimmed sequences were aligned with the human reference genome (hg38.13) using Bowtie2^50^ and removed the duplicates using the Bismark v.0.22.3^51^. The methylation extractor from Bismark was used to extract the CpG methylation data from the sequences. The SAMtools (v1.7)^52^ package was used to sort the bam files on CpG-site chromosomal location and converted to SAM files. The methylated and unmethylated CpG counts were extracted and combined using the DMRfinder (v0.3)^53^ package in R (v4.0.2)^54^.

To read the Bismark coverage files, the *edgeR* (v3.32.1)^55,56^ package was used. The CpG-sites located in the X and Y chromosome as well as CpG-sites from mitochondrial DNA were filtered out. CpG-sites with a read coverage < 5 with both methylated and unmethylated reads were removed from the analysis. The M-values were calculated using the log_2_ ratio of the intensities of methylated verses unmethylated CpG-sites, Eq. (1) (with addition of + 2 to each count to avoid logarithms of zeros)^57^.

2 $$M_{i} = log_{2} \left( {\frac{{\max \left( {y_{i, methylation,} 0} \right) + \alpha }}{{\max \left( {y_{i, unmethylation,} 0} \right) + \alpha }}} \right),\quad \alpha = 2$$

The CpG-sites were annotated using *org.Hs.eg.db* (v3.12)^58^ and AnnotationDbi (v1.52)^59^ packages using human genome hg38. After filtering and annotating the data, we identified a total of 1,186 CpG-sites in the AMs and 404 CpG-sites from alveolar T cells that were covered in all samples.

### Statistical analysis

The Principal Component Analysis (PCA) was calculated using the *factoExtra* (v1.0.7)^60^ and *factoMineR* (v2.4)^61^. To examine the correlation of DNA methylation and regulation of gene expression, the Spearman’s rank correlation coefficient was calculated between the scaled M-values^62^ and normalized expression values for the 1,186 CpG-sites and corresponding genes using ggcorr function in GGally package in R^63^. Heatmaps were generated using ComplexHeatmap package (v 4.0.3). To determine which confounding variables or known sources of variation explain the total variance of the data set, we applied principal component analysis (PCA) and calculated the correlation (R^2^) of PCs of the independent variables (IGRA status, gender, age, BMI, batch and timepoint of sample collection). The difference of the variable BMI between the groups was calculated using Mann–Whitney U test in GraphPad Prism 9. The hierarchical clusters was estimated using the *ape* (v5.4-1)^64^ and *dendextend* (v1.14.0)^65^ packages by calculating the Euclidian similarity/dissimilarity matrix. For the identification of the individual differentially methylated CpG-sites (DMCs) the EdgeR *lmFit* function^66^ was used. Identifying differentially methylated genes in the same study subject longitudinally instead of comparing groups reduces the risk of confounding and is suitable with a small sample size. The counts of the unmethylated (Un) and methylated (Me) reads in the conditions 0 months (A) and 6 months (B) in each individual were used to calculate the Fold Change, Eq. (2). The dispersion parameter controlling the degree of biological variability was set to 0.0247.

3 $$\begin{aligned} & \beta_{A} = { }log_{2} \left( {\frac{{{\text{MeA}} + { }}}{{{\text{UnA}} + { }}} } \right) ,\quad \alpha = \, 0.{125} \\ & \beta_{B} = { }log_{2} \left( {\frac{{{\text{MeB}} + { }}}{{{\text{UnB}} + { }}} } \right) ,\quad \alpha = \, 0.{125} \\ & |log_{2} {\text{FC}}| = { }\beta_{B} - \beta_{A} \\ \end{aligned}$$

DMCs were defined as CpG-sites with a |log_2_FC|> 5 and significant with the BH adjusted *p* value < 0.01. The lollipop plots and the upset plots were made using the *ggpubr* (v0.4.0)^67^ and the *UpSetR* (v1.4.0)^68,69^ packages. Two IGRA_neg_ study subjects and 1 study subject had no DMCs with a |log_2_FC|> 15 in the AMs and alveolar T cells respectively and were therefore excluded from the analysis. The pathway analysis was performed with the DMGs with a |log_2_FC|> 15 and BH adjusted *p *value < 0.01 unique to the IGRA converters AMs and alveolar T cells respectively from the PANTHER database (v16.0)^24^ using the WEB-based Gene SeT AnaLysis Toolkit (WebGestalt) webserver (v2019)^23^. The false discovery rate (FDR) in the pathway analysis is BH adjusted *p *values. The significant pathways identified in the AMs are presented in Table 2 and the top 4 most significant pathways identified in the alveolar T cells are presented in Table 3.

## Supplementary Information

Supplementary Information 1.

Supplementary Information 2.

Supplementary Information 3.

Supplementary Information 4.

Supplementary Legends.

## Data Availability

The datasets will be made available at the final publication.
