# Supplementary material for: A differential DNA methylome signature of pulmonary immune cells from individuals converting to latent tuberculosis infection
Source: Sci Rep. 2021 Sep 30;11:19418. doi: 10.1038/s41598-021-98542-3 (PMC8484443; doi:10.1038/s41598-021-98542-3)
Supplement: Supplementary file 4 — Supplementary Information 4. [file 41598_2021_98542_MOESM4_ESM.docx]

Table S1

|  | 0 months | | 6 months | |
| --- | --- | --- | --- | --- |
|  | **TB1-Nil** | **TB2-Nil** | **TB1-Nil** | **TB2-Nil** |
| ***Pos 1*** | 0.33 | 0.52 | - | - |
| ***Conv 1*** | 0.03 | 0.06 | 2.43 | 2.28 |
| ***Conv 2*** | -0.01 | 0 | 6.62 | 6.8 |
