# Supplementary material for: A differential DNA methylome signature of pulmonary immune cells from individuals converting to latent tuberculosis infection
Source: Sci Rep. 2021 Sep 30;11:19418. doi: 10.1038/s41598-021-98542-3 (PMC8484443; doi:10.1038/s41598-021-98542-3)
Supplement: Supplementary file 5 — Supplementary Legends. [file 41598_2021_98542_MOESM5_ESM.docx]

Figure S1. **Correlation of transcriptome and DNA methylation**. **A.** A heatmap showing the scaled M-value (methylome) and gene count (transcriptome) for 1,186 genes in 6 samples (3 donors with samples collected at two timepoints). The legend shows the scale of the data.

**B**. A correlation matrix showing the negative Spearman’s rank correlation coefficients (ranging from -1 to 1) between the methylation and transcriptome.

Figure S2. **Correlation plot of principal components and confounding variables.** Variance decomposition of the principal components illustrating R^2^ value of confounding variables at the principal components in the data for A. Macrophages and B. T cells. C. A Mann-Whitney U test comparing the BMI of study participants between the groups IGRA-converters and IGRA-negatives showing no significant difference (p value = 0.89).

Figure S3. **UpSet plot showing the intersects of the DMGs identified in each study subject’s alveolar T cells.** UpSet plot showing the intersects of the differentially methylated genes (DMGs) identified in each study subjects’ alveolar T cells.
